# Tricin Biosynthesis and Bioengineering

**DOI:** 10.3389/fpls.2021.733198

**Published:** 2021-08-26

**Authors:** Pui Ying Lam, Andy C. W. Lui, Lanxiang Wang, Hongjia Liu, Toshiaki Umezawa, Yuki Tobimatsu, Clive Lo

**Affiliations:** ^1^Research Institute for Sustainable Humanosphere, Kyoto University, Kyoto, Japan; ^2^School of Biological Sciences, The University of Hong Kong, Pokfulam, Hong Kong, China; ^3^CAS Key Laboratory of Quantitative Engineering Biology, Shenzhen Institute of Synthetic Biology, Shenzhen Institutes of Advanced Technology, Chinese Academy of Sciences, Shenzhen, China; ^4^State Key Laboratory for Managing Biotic and Chemical Threats to the Quality and Safety of Agro-products, Zhejiang Academy of Agricultural Sciences, Hangzhou, China

**Keywords:** tricin, biosynthetic pathways, flavonoids, lignin, bioengineering, biorefinery

## Abstract

Tricin (3',5'-dimethoxyflavone) is a specialized metabolite which not only confers stress tolerance and involves in defense responses in plants but also represents a promising nutraceutical. Tricin-type metabolites are widely present as soluble tricin *O*-glycosides and tricin-oligolignols in all grass species examined, but only show patchy occurrences in unrelated lineages in dicots. More strikingly, tricin is a lignin monomer in grasses and several other angiosperm species, representing one of the “non-monolignol” lignin monomers identified in nature. The unique biological functions of tricin especially as a lignin monomer have driven the identification and characterization of tricin biosynthetic enzymes in the past decade. This review summarizes the current understanding of tricin biosynthetic pathway in grasses and tricin-accumulating dicots. The characterized and potential enzymes involved in tricin biosynthesis are highlighted along with discussion on the debatable and uncharacterized steps. Finally, current developments of bioengineering on manipulating tricin biosynthesis toward the generation of functional food as well as modifications of lignin for improving biorefinery applications are summarized.

## Introduction

**Figure 1:**
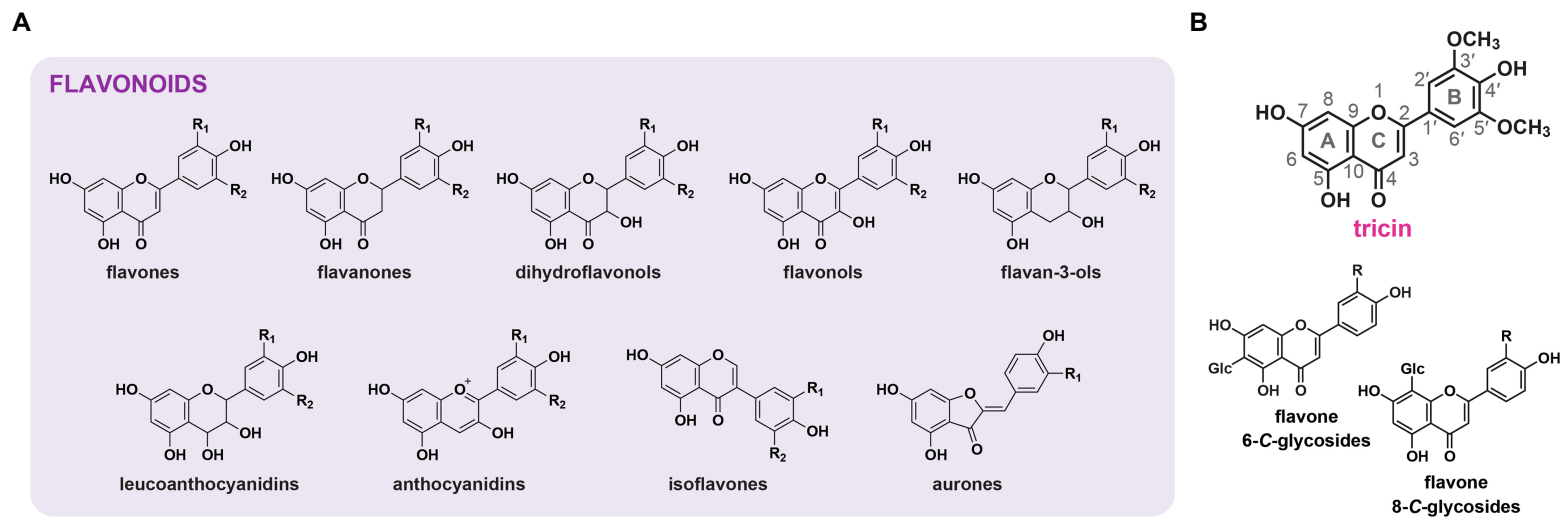
Flavonoids in plants. **(A)** Structures of the major classes of flavonoids in plants. **(B)** Structures of tricin and flavone *C*-glycosides as the major flavone-derived metabolites in grasses. A-, B- and C-rings as well as the numbering system used for flavonoid molecules are indicated. R, R_1_, R_2_: H, OH, or OCH_3_.

Flavonoids are a large group of plant-specialized metabolites that are ubiquitous in vascular plants and are also found in non-vascular plant lineages except hornworts (Yonekura-Sakakibara et al., 2019). Structurally, they are featured by a basic diphenylpropane (C6–C3–C6) backbone, which is usually made up of two benzene rings (A-ring and B-ring) and a middle pyrone ring (C-ring; Alseekh et al., 2020). Flavonoids are assigned to different classes according to the oxidation states in the C-rings (Schijlen et al., 2004). At least nine major classes, namely, flavanones, flavones, dihydroflavonols, flavonols, flavan-3-ols, leucoanthocyanidins, anthocyanidins, isoflavones, and aurones, have been described (Figure 1A; Yang et al., 2018; Nakayama et al., 2019).

In grasses, flavones are the predominant class of flavonoids accumulated in stems and leaves (Harborne and Hall, 1964; Tohge et al., 2017), whereas 3-hydroxylated flavonoids, such as flavonols and anthocyanidins, which are widely distributed in other plant lineages, are usually not accumulated due to the absence of flavanone 3-hydroxylase (*F3H*) expression (Deboo et al., 1995; Shih et al., 2008; Wang et al., 2020b). Grass flavones are present in the forms of flavone *O*-conjugates and flavone *C*-glycosides (Figure 1B; Harborne and Hall, 1964; Brazier-Hicks et al., 2009; Dong et al., 2014). Flavone *O*-conjugates harbor sugar or monolignol moieties linked to flavone aglycones through glycosidic or ether bonds (Li et al., 2016; Lan et al., 2019). 3',5'-Substituted flavone *O*-conjugates, in particular, tricin *O*-conjugates, are widely present (Dong et al., 2014; Li et al., 2016). On the other hand, flavone *C*-glycosides contain sugar moieties directly attached to C6 and/or C8 of the flavone backbones *via* C–C linkages (Besson et al., 1985; Cummins et al., 2006; Brazier-Hicks et al., 2009). Such flavone *C*-glycosides could be 3'-substituted but are rarely 3',5'-substituted (Dong et al., 2014). Unlike flavone *O*-conjugates, flavone *C*-glycosides are resistant to enzymatic or acid hydrolysis.

The flavone tricin has been drawing attention due to its widespread and abundant occurrence as soluble *O*-conjugates in grasses, and more remarkably, its unique incorporation in lignin polymers in cell walls of grasses and some other species. Soluble tricin was first isolated as an aglycone from leaves of a rust-resistant wheat cultivar (*Triticum dicoccum*; cv. Khapli; Anderson and Perkin, 1931). It was later found to be widely distributed in grasses and could also be detected in other monocots (e.g., Cyperaceae members), some dicots (e.g., *Medicago* species), and lycophytes (e.g., *Lycopodium japonicum*) [reviewed by Wollenweber and Dörr, (2008); Zhou and Ibrahim, (2010); Li et al. (2016)]. Soluble tricin-type metabolites usually exist as aglycone or tricin *O*-glycosides (predominately 5-*O*-, 7-*O*- and/or 4'-*O*-glucosides), tricin-oligolignols (predominately 4'-*O*-oligolignols and their derivatives), and their *O*-glycosides [reviewed by Zhou and Ibrahim, (2010); Li et al. (2016); Lan et al., (2019)]. Tricin *C*-glycosides (Theodor et al., 1981; Markham et al., 1987; Peterson and Rieseberg, 1987; Sun et al., 2013b), tricin sulfate, and tricin *O*-glycoside-*O*-sulfates (Harborne, 1975; Harborne and Williams, 1976; Barron et al., 1988; Galland et al., 2014) were also identified. In plants, soluble tricin-type metabolites were reported to function as defensive compounds against fungal pathogens (Kong et al., 2010), weeds (Kong et al., 2004), and insects (Adjei-Afriyie et al., 2000; Bing et al., 2007).

In the last decade, tricin was discovered to be incorporated into lignins (del Río et al., 2012), which are abundant structural polymers deposited together with cellulose and hemicelluloses in secondary cell walls of vascular plants. Tricin-integrated lignin (tricin-lignin; predominately 4'-*O*-conjugated to the β-position of the monolignol-derived phenylpropane units) is extensively distributed in grasses and is also detected in some non-grass monocot species [e.g., coconut (*Cocos nucifera*), curaua (*Ananas erectifolius*), and vanilla (*Vanilla planifolia* and *V*. *phalaenopsis*)] and the dicot alfalfa (*Medicago sativa*; Mao et al., 2013; You et al., 2013; Lan et al., 2016b). Tricin is the first lignin monomer known to be generated outside the monolignol biosynthetic pathways (del Río et al., 2012, 2020; Lan et al., 2015, 2016a, 2019). Currently, the physiological functions of tricin in cell wall lignins remain largely unknown.

To humans, tricin is considered promising nutraceutical due to its anticancer (Hudson et al., 2000; Yue et al., 2020), antioxidant (Ajitha et al., 2012), anti-inflammatory (Shalini et al., 2012, 2016), antiviral (Yazawa et al., 2010; Akuzawa et al., 2011), and antihistaminic activities [reviewed by Zhou and Ibrahim, (2010); Lan et al. (2016, 2019); Jiang et al. (2020)]. The potential use of tricin as a chemopreventive agent was notably well investigated (Hudson et al., 2000; Cai et al., 2004, 2005, 2009; Oyama et al., 2009; Chung et al., 2018; Tanaka et al., 2019; Wu and Tian, 2019; Yue et al., 2020). Tricin has been shown to be suitable for clinical development because of its excellent pharmacological efficacy (Cai et al., 2009) and low toxicity (Verschoyle et al., 2006), whereas its low bioavailability could be overcome by prodrug modifications (Ninomiya et al., 2011).

Elucidating the biosynthetic pathway for tricin is the pre-requisite for genetic manipulation of soluble and lignin-integrated tricin in different biotechnological applications. Here, we delineate the current understandings on tricin biosynthesis and discuss the present development and future prospects regarding the biotechnological aspects of engineering the biosynthetic pathway.

## Tricin Biosynthesis

### Early Biosynthesis – The General Phenylpropanoid Pathway

**Figure 2:**
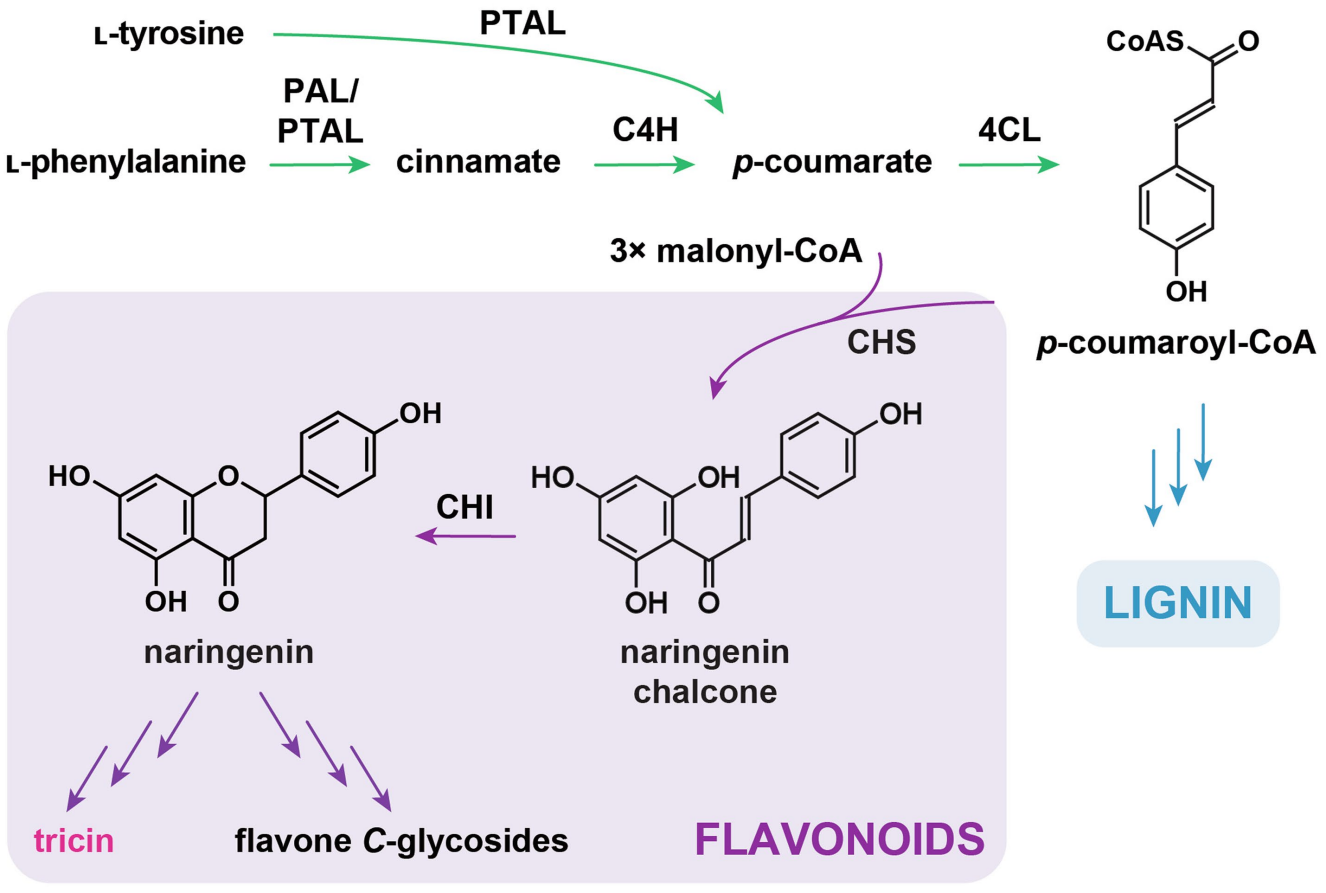
General phenylpropanoid pathway and early flavonoid biosynthetic pathway. PAL, ʟ-phenylalanine ammonia-lyase; PTAL, ʟ-phenylalanine/ʟ-tyrosine ammonia-lyase; C4H, cinnamate 4-hydroxylase; 4CL, 4-hydroxycinnamate:CoA ligase; CHS, chalcone synthase; and CHI, chalcone isomerase. In green: general phenylpropanoid pathway. In purple: flavonoid biosynthetic pathway. In blue: monolignol biosynthetic pathway.

Same as other flavonoids, tricin is a downstream metabolite of the general phenylpropanoid pathway (Figure 2) in which ʟ-phenylalanine is first deaminated into cinnamate by phenylalanine ammonia-lyase (PAL; Camm and Towers, 1973; Elkind et al., 1990), followed by cinnamate 4-hydroxylase (C4H)-catalyzed *para*-hydroxylation of the aromatic ring to form *p*-coumarate (Russell and Conn, 1967; Russell, 1971; Schilmiller et al., 2009). Afterward, 4-coumarate:coenzymeA ligase (4CL) catalyzes the conversion of *p*-coumarate into *p*-coumaroyl-CoA, which serves as the precursor for the biosynthesis of different specialized metabolites, including flavonoids and lignin (Gui et al., 2011; Li et al., 2015). It is long believed that certain 4CL isoforms are specific for flavonoid biosynthesis (Hu et al., 1998; Ehlting et al., 1999; Sun et al., 2013a; Li et al., 2015).

An alternative pathway using ʟ-tyrosine as a substrate to produce phenylpropanoids is also present in grasses (Figure 2; Barros et al., 2016). Bifunctional phenylalanine/tyrosine ammonia-lyases (PTAL) in maize and *Brachypodium distachyon* catalyze the deamination of ʟ-tyrosine to form *p*-coumarate, while at the same time, these enzymes also harbor PAL activities (Rosler et al., 1997; Barros et al., 2016). PALs and PTALs are highly conserved in grasses, suggesting the co-existence of two parallel pathways for phenylpropanoid production in Poaceae (Barros et al., 2016). In addition, results from feeding experiments using ^13^C-labelled ʟ-phenylalanine and ʟ-tyrosine in *B*. *distachyon* have suggested that PTAL is likely to be associated with the generation of grass-specific cell-wall-bound *p*-coumarate units (Barros et al., 2016). It is unknown whether tricin (soluble and lignin-bound) is derived from the PAL and/or PTAL pathway.

### Early Biosynthesis – Flavonoid Skeleton Formation

The initial biosynthetic steps and enzymes for flavonoid skeleton formation are highly conserved in the plant kingdom. Chalcone synthase (CHS), a prototype in the type III polyketide synthase superfamily, catalyzes sequential condensation of three malonyl-CoAs with *p*-coumaroyl-CoA to form naringenin chalcone (Figure 2). Chalcone isomerase (CHI)-catalyzed or occasionally spontaneous isomerization further converts naringenin chalcone into naringenin (a flavanone), which is the first flavonoid structure formed in the biosynthetic pathway. Naringenin is the precursor for all other flavonoids, including tricin. It was shown that deficiency of *CHS*s in maize and rice resulted in depletion in the accumulation of soluble and/or lignin-integrated tricin (Eloy et al., 2017; Wang et al., 2020a). Although it was not examined previously, CHIs are expected to be involved in tricin biosynthesis based on their conserved catalytic functions in the generation of all classes of flavonoids in plants.

### Early Speculation and Recent Demonstration of Separate Pathways for the Biosynthesis of Flavone *O*-Conjugates and Flavone *C*-Glycosides

**Figure 3:**
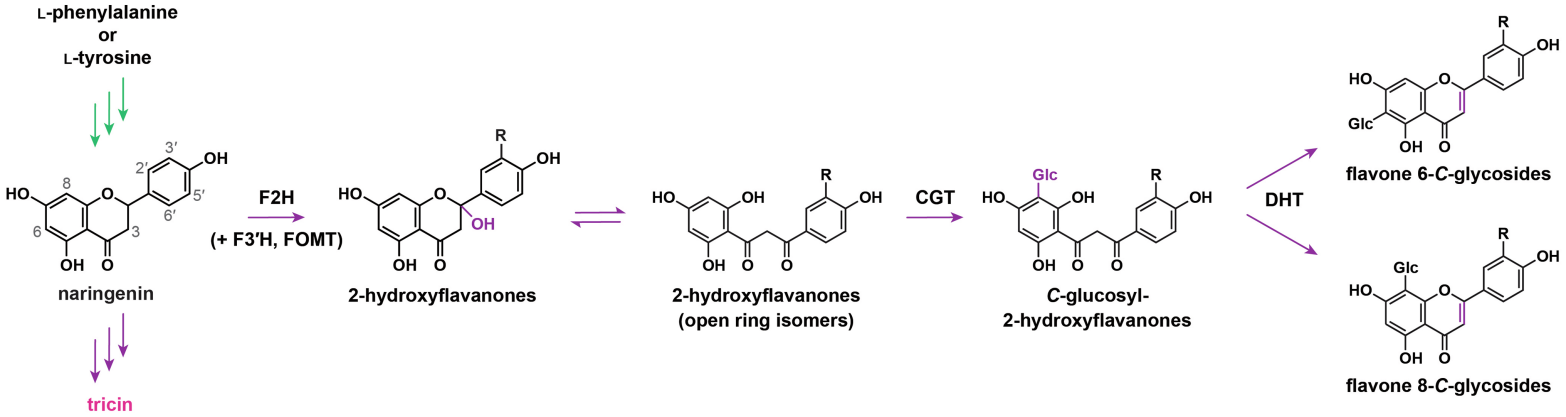
Flavone *C*-glycoside biosynthetic pathway. F2H, flavanone 2-hydroxylase; F3'H, flavonoid 3'-hydroxylase; FOMT, flavonoid *O*-methyltransferase; CGT, *C*-glycosyltransferase; DHT, dehydratase; and Glc, glucose. R: H, OH, or OCH_3_. In green: general phenylpropanoid pathway. In purple: flavonoid biosynthetic pathway.

Flavone *O*-conjugates and flavone *C*-glycosides are biosynthesized in separate pathways. Early radiotracer experiments on Lamnaceae plants revealed that ^14^C-labelled flavanone aglycones could be simultaneously converted into flavone *O*-glycosides and *C*-glycosides (Wallace and Grisebach, 1973), whereas ^14^C-labelled flavone aglycones could only be *O*-glycosylated but could not be *C*-glycosylated (Wallace et al., 1969). Accordingly, it was proposed that *O*-glycosylation occurs at the terminal step after the flavone aglycone is generated, whereas *C*-glycosylation takes place before flavone skeleton formation. Subsequently, crude enzyme extracts prepared from *Fagopyrum esculentum* cotyledons were shown to utilize 2-hydroxyflavanones, instead of flavanones or flavones, as substrates for *C*-glycosylation (Kerscher and Franz, 1987, 1988). These early speculations were substantiated by the characterization of flavone *C*-glycoside biosynthetic pathway in grasses a few decades later. To synthesize flavone *C*-glycosides, flavanones are first converted into 2-hydroxyflavanones by flavanone 2-hydroxylases (F2H; Figure 3), which are cytochrome P450 (CYP) monooxygenases belonging to the subfamily CYP93G (Du et al., 2010; Morohashi et al., 2012). Afterward, 2-hydroxyflavanones or their open ring isomers are *C*-glycosylated by *C*-glucosyltransferase, followed by dehydration to generate the flavone skeletons (Brazier-Hicks et al., 2009; Du et al., 2010; Ferreyra et al., 2013). Meanwhile, it was demonstrated that a rice mutant deficient in *OsF2H* was depleted in the accumulation of various flavone *C*-glycosides, but the production of tricin *O*-conjugates was not affected (Du et al., 2010). Evidently, flavone *O*-conjugates are synthesized in a separate pathway independent from flavone *C*-glycosides.

### Originally Proposed Tricin Biosynthetic Pathway

**Figure 4:**
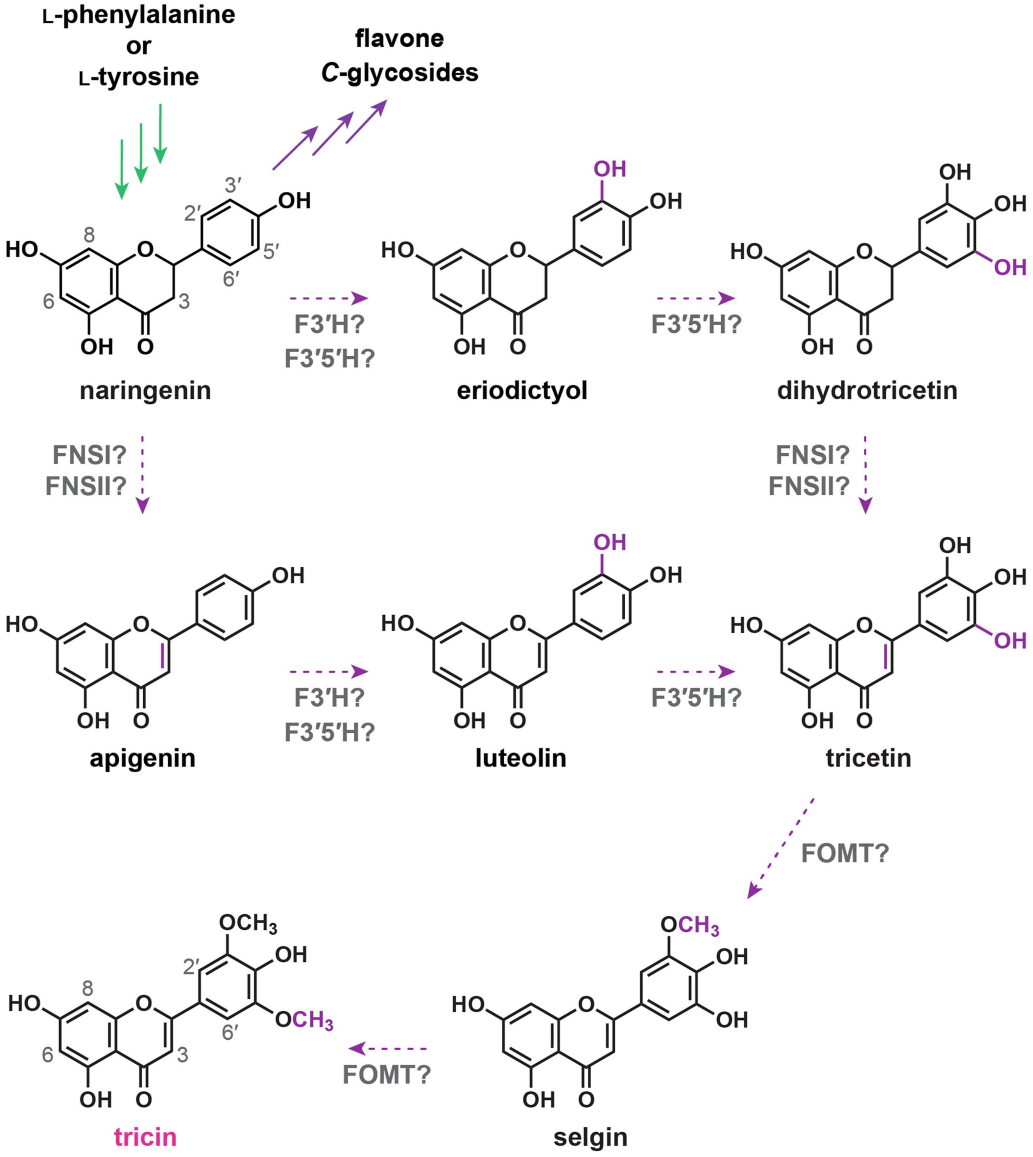
Originally proposed tricin biosynthetic pathway. FNSI, flavone synthase I; FNSII, flavone synthase II; F3'H, flavonoid 3'-hydroxylase; F3'5'H, flavonoid 3',5'-hydroxylase; and FOMT, flavonoid *O*-methyltransferase. In green: general phenylpropanoid pathway. In purple: flavonoid biosynthetic pathway. Dotted arrows: originally proposed tricin biosynthetic pathway.

Structural changes required for converting naringenin into tricin involve desaturation of the C2–C3 bond in the C-ring to generate the flavone nucleus, 3'- and 5'-hydroxylations in the B-ring, and subsequently 3'- and 5'-*O*-methylations. Two different types of enzymes, flavone synthase I (FNSI) and flavone synthase II (FNSII), were expected to convert flavanones into flavones by direct introduction of the C2–C3 double bond (Figure 4). FNSIs are Fe^2+^- and 2-oxoglutarate-requiring soluble enzymes, whereas FNSIIs are CYP enzymes bound to endoplasmic reticulum membranes (Martens and Mithöfer, 2005). Meanwhile, tricetin, a 3',5'-dihydroxylated flavone, was long proposed to be an intermediate along the tricin biosynthetic pathway (Cummins et al., 2006; Zhou and Ibrahim, 2010; Galland et al., 2014). Accordingly, sequential B-ring hydroxylations were expected to be catalyzed by flavonoid 3',5'-hydroxylases (F3'5'Hs). As all known F3'5'Hs accept different classes of flavonoids as substrates, 3',5'-hydroxylations might take place before and/or after flavone formation. Afterward, sequential 3',5'-*O*-methylations of tricetin presumably catalyzed by flavonoid *O*-methyltransferases would occur to produce tricin (Kim et al., 2006; Lin et al., 2006; Zhou et al., 2006, 2008, 2009). Collectively, the reaction steps for tricin biosynthesis were initially proposed to be: naringenin → apigenin → luteolin → tricetin → selgin → tricin (Galland et al., 2014) and/or naringenin → eriodictyol → dihydrotricetin → tricetin → selgin → tricin (Figure 4; Cummins et al., 2006; Zhou and Ibrahim, 2010).

### Current Understanding on Tricin Biosynthesis in Grasses

#### Flavone Nucleus Formation

**Figure 5:**
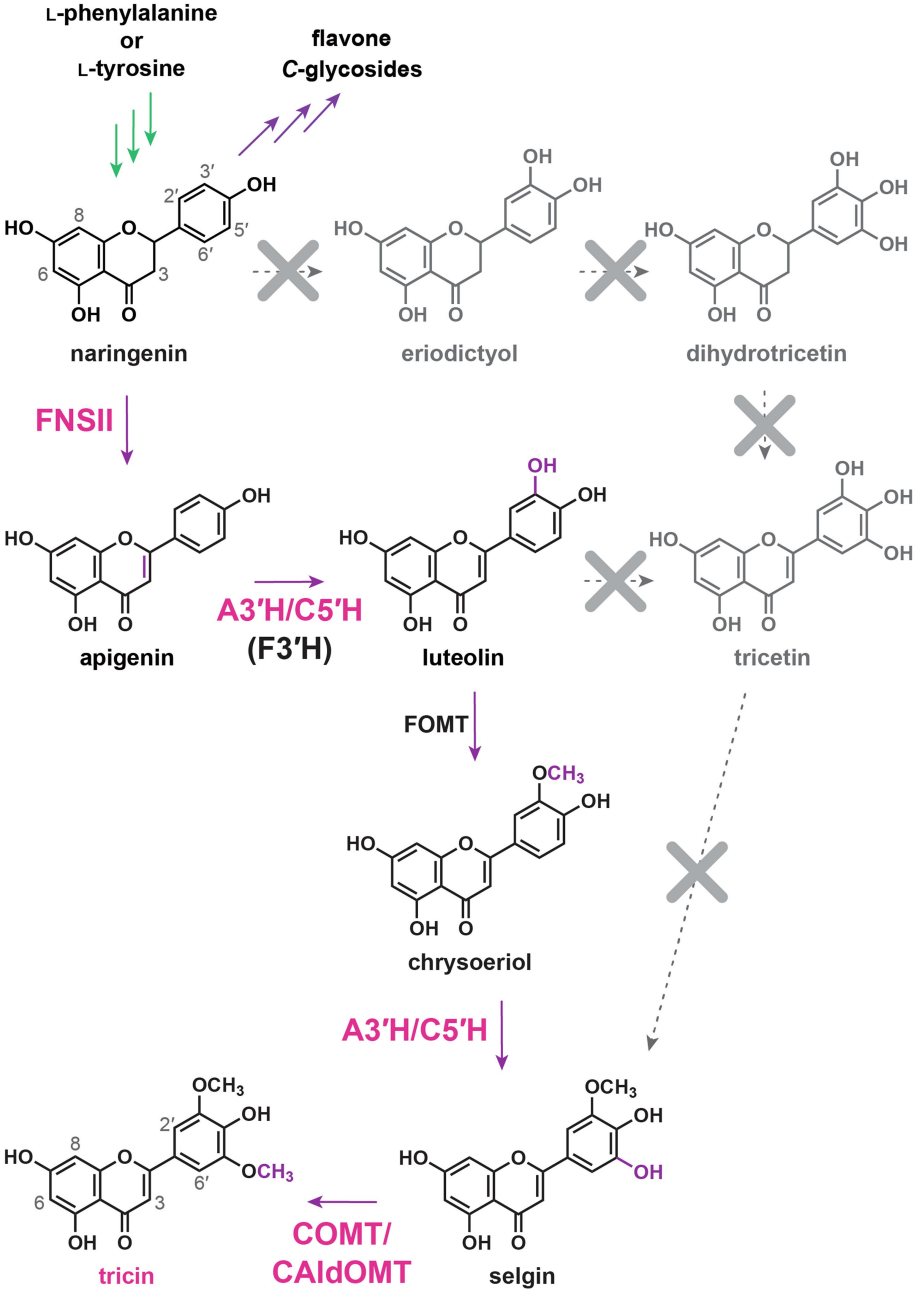
Current understanding on tricin biosynthetic pathway in grasses. FNSII, flavone synthase II; A3'H/C5'H, apigenin 3'-hydroxylase/chrysoeriol 5'-hydroxylase; F3'H, flavonoid 3'-hydroxylase; FOMT, flavonoid *O*-methyltransferase; COMT, caffeic acid *O*-methyltransferases; and CAldOMT, 5-hydroxyconiferaldehyde *O*-methyltransferase. In green: general phenylpropanoid pathway. In purple: flavonoid biosynthetic pathways. Dotted arrows: originally proposed tricin biosynthetic pathway.

Using rice (*Oryza sativa*) as a model system, FNSII was identified to be the primary enzyme generating the flavone nucleus for tricin biosynthesis in grasses (Figure 5; Lam et al., 2014). Recombinant OsFNSII catalyzes direct conversions of flavanones, i.e., naringenin and eriodictyol, into apigenin and luteolin, respectively, *in vitro* (Brazier-Hicks and Edwards, 2013; Lam et al., 2014). In addition, over-expression of *OsFNSII* in Arabidopsis resulted in the accumulation of flavones (apigenin, luteolin, and chrysoeriol) *O*-glycosides which are normally not present in wild-type plants (Lam et al., 2014). Further analyses of the rice *OsFNSII* knockout mutant revealed substantial depletion of soluble tricin *O*-conjugates as well as tricin-lignin in cell walls, demonstrating the direct and predominant involvement of OsFNSII in the generation of both soluble and lignin-integrated tricin in rice (Lam et al., 2014, 2017). Moreover, the *OsFNSII* mutant accumulated soluble naringenin but not the other flavanones, e.g., eriodictyol (Lam et al., 2014), and generated altered lignins incorporated with naringenin (Lam et al., 2017), indicating that the *in planta* substrate of OsFNSII is primarily naringenin.

**Figure 6:**
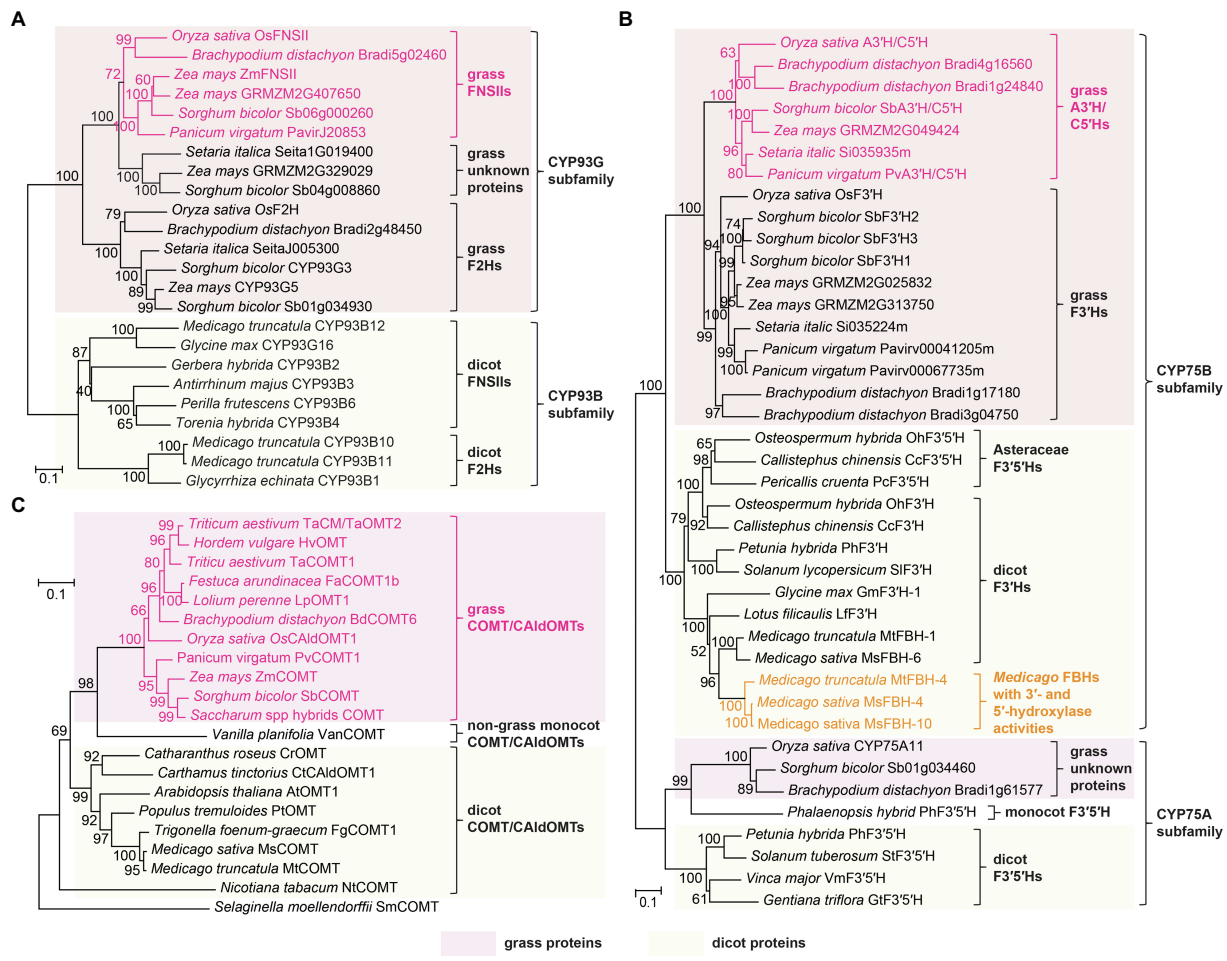
Phylogeny of tricin biosynthetic enzymes. Phylogenetic trees of **(A)** FNSIIs and F2Hs; **(B)** A3'H/C5'Hs, F3'Hs, and F3'5'Hs; and **(C)** COMT/CAldOMTs in grasses and dicots, constructed based on previously published studies (Lam et al., 2017, 2019b; Lui et al., 2020). The unrooted phylogenetic trees were built by neighbor-joining method using MEGAX (Kumar et al., 2018). Bootstrapping with 1,000 replicates was performed. Scale bar denotes 0.1 substitutions per site.

OsFNSII, or CYP93G1, is a P450 enzyme belonging to the same CYP93G subfamily as OsF2H, or CYP93G2. Using naringenin as a common substrate, OsFNSII and OsF2H are the branch-point enzymes for the biosynthesis of tricin *O*-conjugates and flavone *C*-glycosides, respectively (Figures 3, 5). Phylogenetic analysis revealed that OsFNSII and OsF2H form two separate clades, each containing highly conserved sequences from the grass family (Figure 6A; Lam et al., 2017). Hence, sub-functionalization of CYP93G members probably preceded lineage divergence within Poaceae, resulting in the widespread distribution of the two classes of flavone-derived metabolites in grasses today. It is noteworthy that grass FNSIIs and F2Hs have a different phylogenetic origin from dicot FNSIIs and F2Hs, all of which exclusively belong to the CYP93B subfamily (Figure 6A; Kitada et al., 2001; Martens and Mithöfer, 2005; Zhang et al., 2007; Fliegmann et al., 2010; Wu et al., 2016; Zhao et al., 2016; Jiang et al., 2019). Noteworthily, grass species do not contain any CYP93B members and dicots do not have CYP93G members (Du et al., 2016).

Functionally, redundant enzymes other than FNSII are likely to be involved in tricin biosynthesis in grasses. For example, the rice *OsFNSII* mutant still accumulated soluble tricin and other flavones in anthers albeit at reduced levels compared with wild type (Wang et al., 2020a), while it shows substantial depletion of soluble tricin *O*-conjugates and tricin-lignin in vegetative tissues (Lam et al., 2014, 2017). In fact, two rice FNSIs were shown to catalyze the conversion of naringenin into apigenin *in vitro* (Kim et al., 2008; Lee et al., 2008b). In addition, maize possesses an FNSI (ZmFNSI-1) which shows *in vitro* FNS activities and results in the accumulation of flavones when over-expressed in Arabidopsis (Ferreyra et al., 2015; Righini et al., 2019).

#### B-Ring Hydroxylations

In the plant kingdom, 3',5'-substituted flavonoids are patchily distributed, because F3'5'Hs, the enzymes responsible for catalyzing 5'-hydroxylation, are only present in isolated plant lineages (Tanaka and Brugliera, 2013). This is in contrast to the ubiquitous nature of flavonoid 3'-hydroxylases (F3'H; exclusively members of the CYP75B subfamily) that gives rise to the prevalence of 3'-substituted flavonoids (Tanaka and Brugliera, 2013). There have been strong interests for the investigation of F3'5'Hs as they are the key enzymes for the generation of delphinidin-derived anthocyanins, which confer blue or violet coloration in plant tissues, such as flowers and fruits (Tanaka and Brugliera, 2013). For ornamental purposes, transgenic expression of foreign *F3'5'H*s has been employed to engineer novel blue or violet color in roses (*Rosa hybrida*), chrysanthemums (*chrysanthemum morifolium*), and carnations (*Dianthus caryophyllus*), all of which naturally lack delphinidin-derived anthocyanins (Katsumoto et al., 2007; Brugliera et al., 2013; Noda et al., 2013; Tanaka and Brugliera, 2013).

The canonical F3'5'Hs are CYP enzymes belonging to the CYP75A subfamily (Tanaka and Brugliera, 2013). Apparently, CYP75A-encoding genes have been lost repeatedly or became non-functional in many lineages during evolution (Tanaka and Brugliera, 2013). In rice, the only CYP75A member (CYP75A11) did not show any F3'5'H functions in *in vitro* enzyme assays or in *CYP75A11* over-expressing transgenic Arabidopsis plants (Lam et al., 2015). On the other hand, a rice CYP75B member (CYP75B4) solely contributes to the 5'-hydroxylation activity during tricin biosynthesis, as evidenced by *in planta* metabolite analysis. For example, the *CYP75B4* T-DNA knockout mutant is completely devoid of soluble selgin and tricin *O*-conjugates in vegetative tissues (Lam et al., 2015) and tricin-lignin in cell walls (Lam et al., 2019a). In addition, transgenic Arabidopsis co-expressing *CYP75B4* and *OsFNSII* accumulates *O*-conjugates of selgin and tricin (Lam et al., 2015). Meanwhile, apigenin produced by OsFNSII using naringenin as a preferred *in planta* substrate was initially expected to undergo sequential B-ring hydroxylations to form tricetin as a tricin precursor. However, while CYP75B4 3'-hydroxylates apigenin to luteolin, it fails to 5'-hydroxylate luteolin to tricetin (Lam et al., 2015). Instead, CYP75B4 catalyzes 5'-hydroxylation of chrysoeriol to produce selgin (Lam et al., 2015). Chrysoeriol could be generated by 3'-*O*-methylation of luteolin, whereas selgin could undergo 5'-*O*-methylation to generate tricin. The flavonoid B-ring *O*-methylation reactions are known to be catalyzed by several *O*-methyltransferases in rice (see B-ring O-methylations below). Collectively, tricin biosynthetic pathway in rice has been re-established as: naringenin → apigenin → luteolin → chrysoeriol → selgin → tricin (Figure 5; Lam et al., 2015, 2019a). Meanwhile, chrysoeriol *O*-linked derivatives accumulates in rice vegetative tissues (Galland et al., 2014; Lam et al., 2015, 2019a; Eloy et al., 2017), whereas tricetin and its *O*-linked derivatives (e.g., *O*-conjugates) are rarely detected in grasses (Galland et al., 2014; Lam et al., 2015, 2019a; Eloy et al., 2017), supporting that chrysoeriol, instead of tricetin, is an intermediate along the tricin biosynthetic pathway.

CYP75B4 is a flavone-specific bifunctional B-ring hydroxylase in rice. It displays very weak 3'-hydroxylase activity toward naringenin while converting apigenin to luteolin readily (Lam et al., 2015; Park et al., 2016). In addition, its 5'-hydroxylation activity was restricted to chrysoeriol, but not any other 3'-methoxylated or 3'-hydroxylated flavonoids (Lam et al., 2015). Hence, the enzyme is now dedicated as apigenin 3'-hydroxylase/chrysoeriol 5'-hydroxylase (A3'H/C5'H). The dual catalytic activities have also been demonstrated in the highly conserved orthologs in sorghum (CYP75B97) and switchgrass (CYP75B11; Figure 6B), indicating that similar enzymology and intermediates were recruited for tricin biosynthesis in the grass family (Lam et al., 2019a). Further evidence indicated that the 3'-hydroxylation reaction (apigenin → luteolin) for tricin biosynthesis is also predominantly contributed by A3'H/C5'H. Thus, the rice *CYP75B4* mutant accumulates elevated amounts of soluble apigenin metabolites along with the incorporation of apigenin into cell wall lignins (Lam et al., 2015, 2019a). On the other hand, CYP75B3, the only other CYP75B member in rice, is a canonical F3'H which catalyzes *in vitro* 3'-hydroxylation of a wide range of flavonoids including apigenin (Shih et al., 2008; Lam et al., 2015, 2019a; Park et al., 2016). However, *CYP75B3* loss-of-function mutants are preferentially deficient in 3'-substituted flavone (luteolin and chrysoeriol) *C*-glycosides, while their production of soluble and lignin-integrated tricin remains unaffected (Lam et al., 2019a). Apparently, CYP75B3 primarily functions together with OsF2H along the separate biosynthetic pathway for flavone *C*-glycosides (Figure 3).

The highly conserved A3'H/C5'Hs in grasses are distinctive from other F3'5'Hs with regard to their phylogeny and catalytic properties. They are phylogenetically distant from CYP75A F3'5'Hs and were likely recruited through neofunctionalization of an ancestral CYP75B F3'H protein (Figure 6B). Similarly, several Asteraceae species had acquired CYP75B F3'5'Hs independently through convergent evolution, leading to delphinidin-derived anthocyanin pigmentation (Seitz et al., 2006; Seitz et al., 2015). In addition, the grass A3'H/C5'Hs are substrate specific for both 3'-hydroxylation (apigenin) and 5'-hydroxylation (chrysoeriol), while CYP75A and Asteraceae CYP75B F3'5'Hs could utilize a variety of non-substituted, 3'-hydroxylated and 3'-methoxylated flavonoids as substrates. Intriguingly, the unique catalytic properties of A3'H/C5'Hs are reminiscent of the bifunctional phenylpropanoid *meta*-hydroxylase (CYP788A1) required for syringyl (S)-lignin biosynthesis in the spikemoss *Selaginella moellendorffii*. CYP788A1 is involved in both 3- and 5-hydroxylations of phenylpropanoids, but it could only catalyze 5-hydroxylation after 3-*O*-methylation (Weng et al., 2008, 2010).

#### B-Ring *O*-Methylations

**Figure 7:**
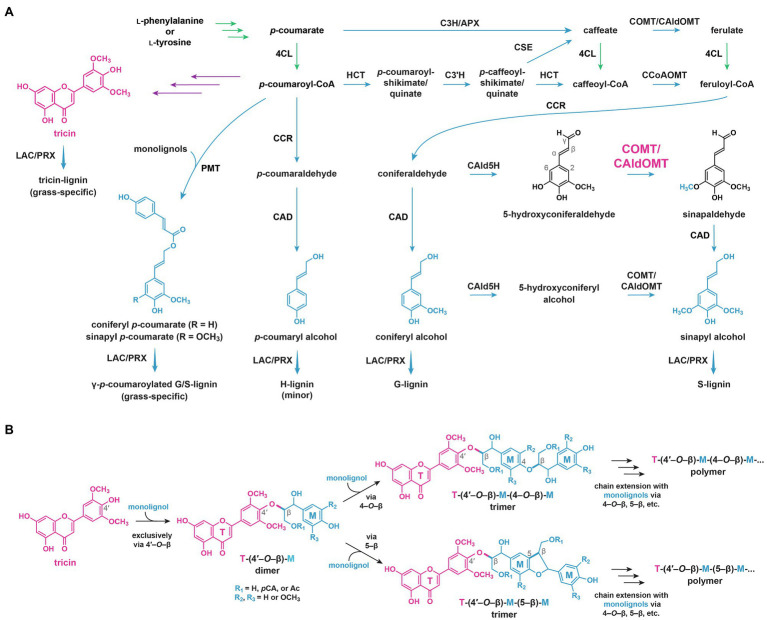
Current understanding on lignin biosynthesis in grasses. **(A)** Monolignol biosynthetic pathways. Major activity of COMT/CAldOMT in monolignol biosynthetic pathways is indicated. **(B)** Radical coupling of tricin and monolignols to produce tricin-lignin polymers. 4CL, 4-hydroxycinnamate:CoA ligase; C3H, *p*-coumarate 3-hydroxylase; HCT, *p*-hydroxycinnamoyl-CoA:quinate/shikimate esterase; APX, ascorbate peroxidase; C3'H, *p*-coumaroyl ester 3-hydroxylase; CSE, caffeoyl shikimate esterase; CCoAOMT, caffeoyl-CoA *O*-methyltransferase; CAld5H, coniferaldehyde 5-hydroxylase; COMT, caffeic acid *O*-methyltransferases; CAldOMT, 5-hydroxyconiferaldehyde *O*-methyltransferase; CCR, cinnamoyl-CoA reductase; CAD, cinnamyl alcohol dehydrogenase; PMT, *p*-coumaroyl-CoA:monolignol transferase; LAC, laccase; and PRX, peroxidase. In green: general phenylpropanoid pathway. In purple: flavonoid biosynthetic pathways. In blue: monolignol biosynthetic pathways. T, tricin; M, monolignols and their derivatives; *p*CA, *p*-coumarate; and Ac, acetate.

Several cation-independent OMTs in grasses were found to catalyze *in vitro O*-methylation of flavones in grasses (Kim et al., 2006; Lin et al., 2006; Zhou et al., 2006, 2008, 2009). Interestingly, these enzymes have been annotated as caffeic acid *O*-methyltransferases (COMT) or 5-hydroxyconiferaldehyde *O*-methyltransferases (CAldOMT) as they also show *in vitro O*-methylation activities toward 5-hydroxyconiferaldehyde, 5-hydroxyferulic acid, and caffeic acid, which are intermediates in the monolignol pathway; hence, they are also involved in S-lignin biosynthesis (Figure 7A; Collazo et al., 1992; Piquemal et al., 2002; Ma and Xu, 2008; Sattler et al., 2012; Koshiba et al., 2013).

Recently, knockout and knockdown mutant analyses have demonstrated that grass COMT/CAldOMTs are actually bifunctional enzymes required for both tricin and S-lignin biosynthesis (Fornalé et al., 2016; Eudes et al., 2017; Daly et al., 2019; Lam et al., 2019b). Rice and sorghum deficient in *COMT/CAldOMT* accumulated reduced levels of soluble tricin but increased levels of selgin (mono-methoxylated) and luteolin (non-methoxylated) when compared with wild-type controls (Lam et al., 2015; Eudes et al., 2017). In addition, maize, rice, and sorghum plants deficient in *COMT/CAldOMT* were depleted in both tricin-lignin and S-lignin (Fornalé et al., 2016; Eudes et al., 2017; Lam et al., 2019b). Apparently, the highly conserved grass COMT/CAldOMT orthologs (Figure 6C) have likely evolved dual catalytic functions for the two parallel biosynthetic pathways of flavonoids and monolignols, contributing to the widespread occurrence of soluble and lignin-integrated tricin metabolites in the grass family nowadays.

Based on the revised tricin biosynthetic pathways and the new findings in the *COMT/CAldOMT*-deficient grass plants, the catalytic activities of COMT/CAldOMTs were re-examined. Recombinant COMT/CAldOMTs in rice and sorghum were found to catalyze 3'-*O*-methylation of luteolin and 5'-*O*-methylation of selgin (Kim et al., 2006; Lin et al., 2006; Zhou et al., 2006; Eudes et al., 2017; Lam et al., 2019b), which are the substrates of COMT/CAldOMTs in the tricin biosynthetic pathway (Figure 5). Meanwhile, rice OsCAldOMT1 shows comparable catalytic efficiencies toward selgin and 5-hydroxyconiferaldehyde, which are the substrates of COMT/CAldOMTs in tricin and monolignol biosynthetic pathway, respectively (Figures 5, 7A), further suggesting the bifunctional roles of COMT/CAldOMTs in tricin and monolignol biosynthesis in grasses (Lam et al., 2019b).

Functionally redundant OMTs other than COMT/CAldOMTs appear to be present for the biosynthesis of tricin in grasses as tricin-derived metabolites, including tricin-lignin, are not completed depleted in the *COMT/CAldOMT* loss-of-function mutants in maize, sorghum, and rice (Lam et al., 2015, 2019b; Fornalé et al., 2016; Eudes et al., 2017). In fact, several cation-dependent caffeoyl-CoA *O*-methyltransferase (CCoAOMT)-related enzymes could catalyze 3',5'-*O*-methylation using various flavone substrates (Lee et al., 2008a), but their involvement in tricin biosynthesis *in planta* requires further investigations.

#### Further *O*-Conjugations After Tricin Formation

Based on the types of soluble tricin metabolites detected in grasses, *O*-glycosylations and *O*-conjugations with monolignols and their acylated derivatives represent the predominant structural modifications of tricin (Dong et al., 2014; Lan et al., 2016a; Eloy et al., 2017; Peng et al., 2017). These modifications occur after the formation of tricin aglycone (Hong et al., 2007; Jiang et al., 2016; Lan et al., 2016a).

*O*-Glycosylations of flavonoids are usually catalyzed by uridine diphosphate (UDP)-dependent glycosyltransferases (UGT; family 1 glycosyltransferases 1; GT1; Ko et al., 2006; Yonekura-Sakakibara and Hanada, 2011; Kim et al., 2015), which utilize UDP sugars as sugar donors (Yang et al., 2018). A number of UGTs from rice (Ko et al., 2006, 2008; Hong et al., 2007; Luang et al., 2013; Chen et al., 2014; Peng et al., 2017) and wheat (Shi et al., 2020) are capable of catalyzing the conjugation of sugars, usually glucose, to one or multiple hydroxyl groups of tricin *in vitro* and/or when over-expressed in transgenic plants. Single-nucleotide polymorphisms (SNPs) in several putative UGTs were also found to be directly associated with the variations of flavone *O*-glycoside accumulation in different natural cultivars and/or recombinant inbred lines of rice (Chen et al., 2014; Dong et al., 2014; Peng et al., 2017; Li et al., 2019) and wheat (Shi et al., 2020). The different *O*-glycosylations could enhance solubility and stability, and might be involved in regulating storage, transport, and detoxification of tricin (Yonekura-Sakakibara and Hanada, 2011).

**Figure 8:**
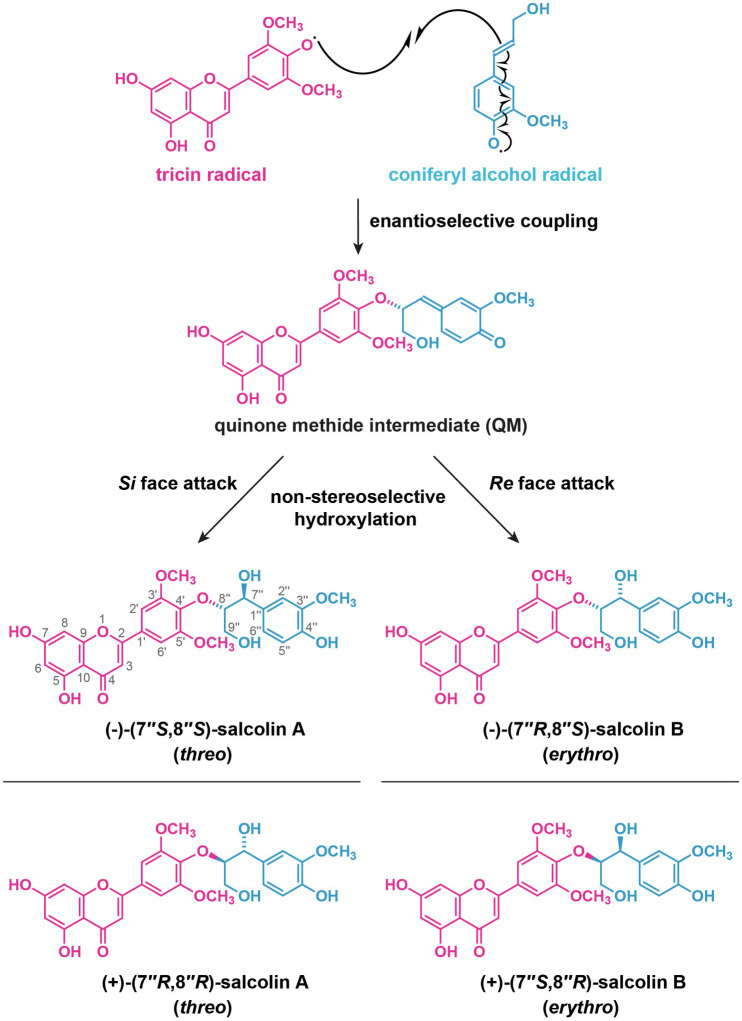
A possible mechanism for the formation of optically active salcolin A and salcolin B through radical coupling of tricin and coniferyl alcohol. Different possible stereoisomers of salcolin A and salcolin B are shown.

In addition to sugars, tricin conjugates with monolignols and their derivatives, leading to the formation of soluble tricin-oligolignols along with insoluble tricin-lignin in the cell walls. The soluble tricin-oligolignols in grasses have been found to be either optically active (Wenzig et al., 2005; Xiong et al., 2011) or inactive (racemic; Lan et al., 2016a). The optically active tricin-oligolignols, which have been often referred to as “flavonolignans” (Begum et al., 2010; Chambers et al., 2015; Csupor et al., 2016), may be formed by oxidative radical coupling of tricin with monolignols or their derivatives with the assistance of dirigent proteins, similar to the biosynthesis of lignans (Davin and Lewis, 2003; Umezawa, 2003; Paniagua et al., 2017), in which dirigent proteins serve as auxiliary proteins for guiding the regioselective and stereoselective coupling of phenoxy radicals from monolignols and their analogs. For example, the absolute configuration of a diastereomeric pair of β–*O*–4 neolignan-type flavonolignans, *threo*-(−)-guaiacylglycerol-β-tricin ether [(−)-salcolin A], and *erythro*-(−)-guaiacylglycerol-β-tricin ether [(−)-salcolin B] isolated from *Sinocalamus affinis* (Poaceae) were determined as 7''*S*,8''*S* and 7''*R*,8''*S*, respectively (Xiong et al., 2011). This strongly suggests that the coupling between tricin and coniferyl alcohol radicals to form 4'–*O*–8'' bond proceeds enantioselectively, probably mediated by a dirigent protein, giving rise to the optically active quinonemetide, which are then attacked by water non-stereoselectively, giving rise to both (−)-(7''*S*,8''*S*)-salcolin A and (−)-(7''*R*,8''*S*)-salcolin B (Figure 8). This is in line with the recent findings that a dirigent protein, AtDIR12/AtDP1, was involved in the formation of arylglycerol-β-aryl ether (β–*O*–4) type neolignans in Arabidopsis (Yonekura-Sakakibara et al., 2021). However, from *Avena sativa*, (−)-salcolin A and (+)-salcolin B were isolated (Wenzig et al., 2005). In this case, the diastereomers should have opposite absolute configuration at 8'' position, forming (−)-(7''*S*,8''*S*)-salcolin A and (+)-(7''*S*,8''*R*)-salcolin B (Figure 8). During their formation, the radical coupling should afford racemic quinonemethide in terms of 8'' position, and the following water addition at 7'' position should be diastereoselective to give rise to the optically active diastereomers (Wenzig et al., 2005). On the other hand, optically inactive tricin-oligolignols are generated solely by radical coupling (Figure 7B) and are considered to exist at least partially as the precursors for the generation of tricin-lignin polymers (see Tricin-lignin formation below; Lan et al., 2016a).

#### Tricin-Lignin Formation

Tricin is incorporated into lignin polymers in grass cell walls by radical coupling (Lan et al., 2015), essentially the same way lignification takes place solely with monolignols (coniferyl alcohol, sinapyl alcohol, and *p*-coumaryl alcohol) in typical non-grass vascular plants (i.e., gymnosperms, dicots, and non-grass monocots). The compatibility of tricin with radical coupling was demonstrated by biomimetic oxidations of tricin with monolignols using peroxidase/hydrogen peroxide and silver (I) oxide as oxidants (Lan et al., 2015). Tricin was found to cross-couple to monolignols exclusively *via* the 4'–*O*–β-coupling mode (Figure 7B; del Río et al., 2012; Lan et al., 2015), probably because the radical from the 4'-hydroxyl group of tricin is more stabilized than the other possible radicals as supported by a density functional theory study (Elder et al., 2020). Thus, in plant cell walls, it is expected that tricin is first oxidized by phenol oxidases, presumably laccases (LAC) and/or peroxidases (PRX; Figure 7A; Tobimatsu and Schuetz, 2019), and then coupled with monolignol radicals or acylated monolignol radicals to form tricin-(4'–*O*–β)-linked phenylpropane units in the lignin polymers (Figure 7B). As tricin is unable to undergo dehydrodimerization, and it does not cross-couple directly with growing lignin polymers, tricin predominantly incorporates into the starting ends of the final lignin polymer chains (Lan et al., 2015). Thus, tricin is expected to serve as a nucleation site for lignification (Lan et al., 2015; Berstis et al., 2021).

Lignin-integrated tricin content in grasses was estimated to be around 0.5–7mg/g whole cell wall or 2–33mg/g lignin by thioacidolysis (Lan et al., 2016b). These contents are several folds higher than extractable tricin content (Lan et al., 2016b), suggesting that the majority of tricin synthesized in grasses is incorporated into lignin polymers in cell walls.

### Current Understanding on Tricin Biosynthesis in Dicots

#### Overview

In contrast to their prevalence in grasses and other monocot lineages, tricin-derived metabolites are only sporadically distributed in dicots. Metabolomics studies have reported their occurrences in several dicot lineages, spanning from basal dicots, like individual *Ranunculus* spp. (Li et al., 2005; Aslam et al., 2012), to two core dicot lineages: rosids [e.g., *Agelaea pentagyna* (family: Connaraceae), *Medicago* legumes and *Trigonella foenum-graecum* (family: Fabaceae); Kuwabara et al., 2003], and asterids [e.g., *Artemisia vulgaris* (family: Asteraceae), *Leucas cephalotes* (family: Lamiaceae), and *Lonicera japonica* (family: Caprifoliaceae)] (Miyaichi et al., 2006). Meanwhile, tricin-lignin is only detected in leaves of alfalfa, albeit at much lower quantity than those in grasses (Lan et al., 2016b). Intriguingly, although tricin is restricted to certain dicot lineages, its flavone precursors, including apigenin, luteolin, and/or chrysoeriol, are widely distributed in non-tricin-accumulating dicots (Harborne, 1974). Hence, the occurrences of tricin derivatives are probably resulting from independent and convergent recruitment of novel enzyme activities in those isolated tricin-accumulating dicot lineages.

#### Flavone Nucleus Formation

Three possible types of dicot enzymes, FNSIs, FNSIIs, and F2Hs, have been described for flavone nucleus formation (Martens et al., 2001; Martens and Mithöfer, 2005; Zhang et al., 2007; Ferreyra et al., 2015; Li et al., 2020a), but their contribution to tricin biosynthesis remains elusive in tricin-producing dicots. Both FNSIs (Britsch, 1990; Martens et al., 2001; Miyahisa et al., 2006; Yun et al., 2008) and FNSIIs (Kitada et al., 2001; Fliegmann et al., 2010; Wu et al., 2016; Zhao et al., 2016; Jiang et al., 2019) catalyze direct desaturation of flavanones into flavones, whereas F2Hs converts flavanones to 2-hydroxyflavanones which were proposed to be intermediates for generating the flavone skeleton (Akashi et al., 1998; Zhang et al., 2007).

Initially identified in parsley (*Petroselinum crispum*), FNSIs were long presumed to be confined to Apiaceae (Britsch, 1990; Martens et al., 2001; Yun et al., 2008). However, they were subsequently isolated from other dicots, including Arabidopsis (Ferreyra et al., 2015) and *Morus notabilis* (Li et al., 2020). Interestingly, angiosperm FNSIs outside Apiaceae are apparently phylogenetically unrelated to FNSIs in Apiaceae and non-vascular plants; thus, FNSIs were probably evolved convergently in distant plant lineages (Li et al., 2020). Meanwhile, all the known dicot FNSIIs and F2Hs are CYP enzymes belonging to the CYP93B subfamily (Kitada et al., 2001; Martens and Mithöfer, 2005; Zhang et al., 2007; Fliegmann et al., 2010; Wu et al., 2016; Zhao et al., 2016; Jiang et al., 2019). FNSIIs are present in most flavone-accumulating dicots, such as *Gerbera* hybrids (Martens and Forkmann, 1999), *Lonicera japonica*, *L*. *macranthoides* (Wu et al., 2016), *Glycine max* (Fliegmann et al., 2010; Jiang et al., 2010), *Glycyrrhiza echinate* (Akashi et al., 1999), *Salvia miltiorrhiza* (Deng et al., 2018), and *Scutellaria baicalensis* (Zhao et al., 2016). On the other hand, F2Hs were only reported in a few dicot species, including *G*. *echinata* (Akashi et al., 1998), *Chrysanthemum indicum* (Jiang et al., 2019), and *M*. *truncatula* (Zhang et al., 2007). It remains to be investigated whether FNSI, FNSII, and/or F2H are required for tricin biosynthesis which is restricted to isolated dicot lineages, such as the *Medicago* legumes.

#### B-Ring Hydroxylations

Considerable knowledge about the 3'- and 5'-hydroxylation reactions required for tricin biosynthesis in *Medicago* legumes has come to light recently (Lui et al., 2020). Canonical CYP75A F3'5'Hs are not involved in the B-ring modifications, but instead, a group of *Medicago*-unique CYP75B proteins, including *M*. *truncatula* MtFBH-4 as well as alfalfa (*M*. *sativa*) MsFBH-4 and MsFBH-10, are utilized. In *in vitro* enzyme assays, these CYP proteins catalyze 3'-hydroxylation of different flavonoid classes (flavanone, flavone, and flavonol) and 5'-hydroxylation of their 3'-methoxylated derivatives which include chrysoeriol. Furthermore, apigenin is converted to 3'- and 5'-substituted flavones (i.e., luteolin, chrysoeriol, selgin, and tricin) when these CYP75B proteins are transiently expressed in *Nicotiana benthamiana* leaves. Consistent with these findings, *M*. *truncatula MtFBH-4* knockout mutants are completely depleted in tricin *O*-glycosides, hence establishing an indispensable role of MtFBH-4 in tricin biosynthesis. Basically, the same reaction steps that occur in grasses (Figure 5) have been acquired independently by the *Medicago* legumes to produce tricin.

The *Medicago*-unique CYP75B enzymes required for tricin biosynthesis are distinct from the grass A3'H/C5'Hs with regard to their catalytic properties and phylogenetic origins (Lam et al., 2015; Lui et al., 2020). For example, the 5'-hydroxylase activity is restricted to chrysoeriol for the grass enzymes but is extended to other 3'-methoxylated flavonoids for the *Medicago* enzymes. Interestingly, the Thr-to-Gly substitution in the substrate recognition site 6 domain is critical for these *Medicago* enzymes to catalyze the 5'-hydroxylation reactions (Lui et al., 2020). On the other hand, the equivalent position is replaced by a Leu residue in the grass A3'H/C5'Hs (Lui et al., 2020), but it is unknown whether this could account for their more specific substrate preference for 5'-hydroxylation. Meanwhile, the *Medicago*-unique CYP enzymes have likely acquired the novel 5'-hydroxylase activities through neofunctionalization of redundant CYP75B F3'Hs following the divergence of the *Medicago* genus from other lineages in the legume family (Lui et al., 2020). Convergent evolution of CYP75B F3'5'H had also occurred independently in several Asteraceae lineages for the generation of delphinidin-derived blue/violet pigments (Seitz et al., 2006, 2015). By sharp contrast, A3'H/C5'Hs are highly conserved amongst grasses, consistent with prevalence of tricin in the grass family (Lam et al., 2019a). It would be intriguing to decipher the enzymology and evolution of B-ring hydroxylations for tricin biosynthesis in other isolated dicot lineages.

#### B-Ring *O*-Methylations

The enzymes responsible for the 3'- and 5'-*O*-methylation reactions remain elusive for tricin biosynthesis in dicots. It is possible that they are also COMT/CAldOMT enzymes, as in the case for the grass bifunctional OMTs. In fact, Arabidopsis knockout mutant analyses demonstrated the dual roles of COMT/CAldOMT in the production of monolignols and flavonoids (Do et al., 2007; Tohge et al., 2007; Nakatsubo et al., 2008). However, there is no tricin accumulation in Arabidopsis, presumably due to the absence of F3'5'H enzymes. Meanwhile, the expression of an endogenous *COMT* gene is upregulated in transgenic alfalfa over-expressing the gene encoding *N*-acetylserotonin *O*-methyltransferase (MsASMT1), which catalyzes the final step in melatonin biosynthesis (Cen et al., 2020). In addition to increased melatonin formation, the transgenic alfalfa plants produced elevated amounts of various soluble chrysoeriol- and tricin-derived metabolites (Cen et al., 2020), which might be resulting from increased COMT activities. However, FgCOMT1 isolated from the tricin-accumulating legume fenugreek (*Trigonella foenum-graecum*; Kuwabara et al., 2003) could *O*-methylate 5-hydroxyferulic acid but not quercetin (a 3'-hydroxylated flavonol) or tricetin *in vitro* (Qin et al., 2012). Over-expression of *FgCOMT1* in Arabidopsis *atomt1* knockout mutant only partially restored the accumulation of sinapoyl aldehyde and sinapic acid (intermediates of the monolignol biosynthetic pathway) but not isorhamnetin (a 3'-methoxylated flavonol; Qin et al., 2012).

## Future Perspective: Bioengineering on Manipulating Tricin Biosynthetic Pathway

### Bioengineering for Functional Food

Cereals contribute to more than half of the world population’s daily caloric intake, but the commonly consumed polished grains, which are mainly consisting of endosperms, are poor in phytochemicals and minerals (Awika, 2011). Their consumption as staple food in developing countries is associated with micronutrient malnutrition due to the lack of dietary diversity (Bhullar and Gruissem, 2013). To overcome this problem, biofortification through metabolic engineering has been pursued to introduce different phytochemicals and minerals in endosperms of cereal grains (Bhullar and Gruissem, 2013; Saltzman et al., 2013). As a prime example, golden rice engineered with the β-carotene biosynthetic pathway in endosperm was developed to combat vitamin A deficiency (Ye et al., 2000; Paine et al., 2005; Owens, 2018). Following the success of golden rice, cereal crops that accumulate high contents of iron, zinc, and various carotenoids in the edible endosperm have been developed using genetic engineering (Wirth et al., 2009; Johnson et al., 2011; Saltzman et al., 2013; Blancquaert et al., 2015; Singh et al., 2017; Zhu et al., 2018). Recently, transgenic rice with endosperms fortified with flavonoids, anthocyanins, or stilbenoids was also successfully engineered (Baek et al., 2013; Ogo et al., 2013; Zhu et al., 2017), representing potential functional staple food containing different health-beneficial phenolics.

Although tricin and its derivatives have been characterized with many different health-promoting properties (Cai et al., 2004; Duarte-Almeida et al., 2007; Yazawa et al., 2011; Murayama et al., 2012; Jung et al., 2014, 2015; Lee et al., 2015; Shalini et al., 2016), they are rarely present in human diets. Tricin is abundant in vegetative tissues of grasses but is not present in cereal endosperm due to the absence of expression of genes required for tricin biosynthesis (Ogo et al., 2013). Primary dietary sources of tricin include whole cereal grains such as rice, wheat, oat, and barley, in which small amounts of tricin are preserved in the bran (pericarp, testa, aleurone, and embryo; Poulev et al., 2018, 2019), as well as some grass-derived food products, such as sugarcane juice (Duarte-Almeida et al., 2007) and barley leaf powders (Zeng et al., 2018).

Functional food crops that are fortified with tricin could be generated by engineering the entire biosynthetic pathway in edible tissues. Previously, transgenic rice seeds that accumulate tricin were generated by expression of genes from multiple species encoding rice PAL, rice CHS, parsley FNSI, soybean FNSII, blue viola F3'5'H, and rice COMT/CAldOMT (Ogo et al., 2013). Recent establishment of the endogenous biosynthetic pathways in grasses (Lam et al., 2014, 2015, 2019a) and *Medicago* legumes (Lui et al., 2020) as well as further elucidation of the regulatory mechanism should facilitate more effective metabolic engineering in plants or edible tissues that do not naturally produce tricin-type metabolites.

### Bioengineering for Biorefinery

Grasses show great potential as a source of lignocellulosic biomass. A large amount of lignocellulose is produced annually as agricultural residues from worldwide cultivation of grass grain crops, including maize, wheat, rice, barley, and sorghum, as well as grass sugar crops, such as sugarcane and sweet sorghum. In addition, grass energy crops, such as *Miscanthus*, *Erianthus*, switchgrass, and bamboo, which show notably high biomass productivity, are attractive lignocellulose feedstocks for various biorefinery applications (Tye et al., 2016; Bhatia et al., 2017; Umezawa, 2018; Umezawa et al., 2020). Because of the prominent impacts of lignin on the usability of lignocellulose in both polysaccharide- and lignin-oriented biorefinery applications, bioengineering approaches to control lignin content and structure in grass cell walls have been actively investigated (Umezawa, 2018, 2020; Halpin, 2019; Coomey et al., 2020). However, due to our limited knowledge regarding the biological functions and physicochemical properties of tricin-lignin, it is still uncertain how tricin-lignin influences the usability of grass biomass. Thus far, not much has been examined on the effects of manipulating tricin biosynthesis on the utilization properties of grass biomass for different biorefinery applications.

As tricin could serve as a nucleation site for lignification, reducing the content of tricin used for lignification may result in reduction of lignin content and biomass recalcitrance, which may in turn improve the production of fermentable sugars from biomass in the polysaccharide-oriented biorefinery processes (Halpin, 2019). Indeed, tricin-depleted rice mutants deficient in *FNSII* (Lam et al., 2017) or *A3'H/C5'H* (Lam et al., 2017, 2019a) displayed reduced lignin content and improved cell wall digestibility. In contrast, however, tricin-depleted maize mutant deficient in *CHS* showed increased lignin level and reduced cell wall digestibility in leaves albeit no alteration in either lignin content or cell wall digestibility in stems (Eloy et al., 2017). The altered lignin content in the *CHS*-deficient maize leaf cell walls was attributed at least partially to the consequence of the increased carbon flux toward the branching monolignol biosynthesis pathway upon the blockage of the entry of the flavonoid pathway where CHS plays the major role (Eloy et al., 2017). These studies on tricin-depleted grass mutants implicated that disrupting tricin biosynthetic genes not only impedes the formation of tricin-lignin but also affects the formation of the core lignin polymer units derived from monolignols, although the mechanisms underlying this phenomenon remain unclear. Further manipulations of different tricin biosynthetic genes in different grass species are imperative to determine the precise relationships between tricin, lignin content and composition, and cell wall digestibility in tricin-depleted grasses.

On the other hand, increasing the levels of tricin serving as initiation sites for lignin polymerization would theoretically reduce the molecular weight of the lignin polymers, which may potentially improve the efficiency of lignin deconstruction in the polysaccharide-oriented biorefinery processes (Berstis et al., 2021). A recent computational study determined that the bond strengths of the 4'–*O*–β linkages between the tricin- and monolignol-derived lignin polymer units are comparable to the major β–*O*–4 linkages connecting the internal monolignol-derived lignin polymer units, suggesting that introduction of more tricin units in lignin polymers is unlikely to increase the energy for lignin depolymerization (Berstis et al., 2021). Nonetheless, whether such tricin bioengineering strategy to attenuate lignin molecular weight and depolymerization efficiency requires further exploration.

Meanwhile, grass crops bioengineered toward high tricin-lignin content could bring benefits in the lignin-oriented biorefinery approaches by amplifying the supply of tricin or tricin-derived aromatic chemicals. It has been estimated that large quantity of tricin could be released from grass lignins (Ralph, 2020; del Río et al., 2020). However, challenges ahead include developing technologies for efficient extraction and isolation of tricin from grass lignins to meet the stringent purity specifications as well as industrializing the production with maximized cost effectiveness and minimized environmental impacts. As the most abundant aromatic polymers on Earth, lignin has a great potential to serve as starting materials for sustainable production of bulk or functionalized aromatic chemicals (Ragauskas et al., 2014; Rinaldi et al., 2016; Umezawa et al., 2020). Accordingly, chemical and biochemical approaches to depolymerize lignin into useful low molecular weight aromatic compounds have been extensively pursued (Schutyser et al., 2018; Sun et al., 2018; Renders et al., 2019; Abu-Omar et al., 2021). As these studies have mostly focused on the conversions of the major monolignol-derived phenylpropane units in lignin, the consequences of lignin-integrated tricin units in various catalytic and bio-catalytic lignin depolymerization strategies remain an intriguing subject for further investigations.

## Author Contributions

PL, YT, and CL wrote the manuscript with help from all the other authors. All authors contributed to the article and approved the submitted version.

## Conflict of Interest

The authors declare that the research was conducted in the absence of any commercial or financial relationships that could be construed as a potential conflict of interest.

## Publisher’s Note

All claims expressed in this article are solely those of the authors and do not necessarily represent those of their affiliated organizations, or those of the publisher, the editors and the reviewers. Any product that may be evaluated in this article, or claim that may be made by its manufacturer, is not guaranteed or endorsed by the publisher.
